# An Ishmaelite

**Published:** 1888-08-25

**Authors:** Leslie Keith

**Affiliations:** Author of "The Chilcotes," "Uncle Bob's Niece," "East and West," etc.


### An Ishmaelite.

By Leslie Keith,

Author of "The Chilcotes," "Uncle Bob's Niece," "East and West," etc.

*(Continued from p. 330.)*

### Chapter XX.—The Storm Bursts.

The lending library grew in popularity as the winter days rendered the sauntering about the streets, which is the chief form of entertainment on the long light evenings, a less attainable amusement. The processions of young men and maidens who keep company abate their ardour for walks—surely dreary enough at any time—when the snow descends, and the rain falls, and the winds are boisterous, and it is then that a book which tells about other people's love experiences becomes valuable.

The reading has to be taken along with a good many domestic details, for in Gillespie Street there is but one apartment which serves as dining-room, drawing-room, library, nursery, and kitchen all in one. This may in part account for the comparative unpopularity of what is called solid, as distinguished from frivolous, literature. Even the livelier articles in the *Fortnightly* and the *Nineteenth Century* might not be very easy of digestion if one had to assimilate them in a room where the family wash was drying, the family steak (you will always find the best cuts in Gillespie Street) frizzling on the fire, the baby squalling, and the younger fry quarrelling. If we had to do our reading under like circumstances, we too, might be glad to forget our surroundings in learning how the duke wooed the duchess; how Lady Arabella's boudoir was furnished, and what she wore when she went into that superfine society where dukes and duchesses shine.

"They ought to read history and science, of course," said Barbara, "but I can't make them do it, poor little women. The only history they care about is the history of the emotions, and in every love story they read they see a foreshadowing of their own."

"But I'm afraid there are not Lords of Burleigh for all Gillespie Street," said Fred with a smile.

"We don't want them down here," said Barbara quietly. Her thoughts had gone out to Arthur, and his followed her guiltily. He had not reflected on this interpretation of his words.

"I was thinking of—the books," he stammered awkwardly.

Barbara smiled. "Oh," she said, "we are getting rid of the lords and ladies by degrees; we are getting down to what one shocked reviewer calls 'quite common people'—the people who live in hotels and boarding houses, and even in lodgings. Poor reviewer! Dives in his purple is the only person worth considering in his eyes."

"That is not my experience. I have found more of human kindness and charity here than ever Dives showed me."

"Oh," said Barbara coldly, "I dare say you find us very different; but we have our poor little virtues too."

More than once of late she had used this tone in her speech with him; indeed ever since that occasion on which Nellie's disastrous little visit had been paid.

When Barbara had gone back to her duties that day, after a considerable interval, in which Fred was left to his own bitter reflections, she had worn rather a distant air.

"The lady got safely to her carriage," she said. "I believe she has gone home."

"Miss Barbara," said Fred in a low voice, "I thank you. May I tell you who that lady is, and why I did not wish her to come here?"

"Why should you?" said Barbara with a fine indifference. "She would probably object to my knowing anything about her. I do not feel any particular curiosity; she is not one of us. I dare say you were quite right in telling her that this was no fit place for her."

"If it is not, it is because I am here; because I am no fit associate for anyone whose—whose friendship I am mad enough to covet," he said, but she affected not to hear him.

"Indeed," she said, "you know best. Would you mind handing down that book, the last on the top row? Miss Crossfield is coming for it this afternoon."

For some inscrutable feminine reason she was very cold to him, and for some days she hedged herself about with a constant succession of matrons and maidens, whom she permitted the privilege of doing their reading in the quiet corner by the stove. This was an indulgence Barbara did not allow to many people; but she granted it to one or two favoured men and women of sober behaviour, whose lodgings she knew to be scantily warmed.

Why Barbara was angry is best known to her own sex; also why she repented of her anger, and became speedily ashamed of it, and so much kinder in consequence to her assistant, that even Miss Betsy's sharp eyes took note of the change, though household affairs gave her few opportunities of being in the shop.

Miss Betsy played the part of dragon in the Gillespie Street household. She snubbed and hectored the presumptuous Tucker, and kept him in his place. She had a certain respect, mingled with feminine contempt, for her brother; and a great deal of tolerance, without the respect, for her sister-in-law, who was, in her expressive phraseology, a "sumph"—otherwise, a mass of inert good nature, who could not be expected to look after her girls at that dangerous crisis when lovers began to appear upon the horizon.

Tucker was scarcely worth consideration in this light, but Mr. Eastgate was of another order; and Miss Betsy determined that there should be no "on-goings" between him and Barbara. One day she appeared in the shop with her "seam," and ponderously took up her position where nothing could escape her scrutiny. This behaviour was repeated for many days, the seam being varied by a stocking, but the vigilance remaining unchanged.

Barbara accepted it in amused surprise. What had she and her assistant to say to each other that the whole world might not hear? When they were not serving customers, recommending books, or exchanging battered fiction, or selling modest supplies of writing materials, their few remarks were entirely impersonal. They scarcely soared beyond Miss Betsy's range, even when it was some new volume they had under review, and they neither by word or look gave any shock to her severe views of propriety.

Fred, indeed, rarely spoke unless he was spoken to; he worked indefatigably. He was very grave; the most maidenly fears were soothed by his behaviour. His silence, his reticence about himself, his worn and haggard look of premature age, were scarcely likely to attract a young girl; but Miss Betsy was wise enough to know that a man has only to have certain differences—to be, in short, somewhat of a mystery—to be instantly the hero of a romance. Here was a gentleman, if a fallen one, and here was a young, bright girl thrown into daily companionship with him. Miss Betsy saw danger signals ahead.

"If John Proudie's a fool, there's little call for me to shut my eyes to keep him company," said Miss Betsy; and her eyes, indeed, were very bright and sharp and wide awake as she sat, missing nothing of all that passed around her.

Sometimes Delia joined the working party below, and Miss Betsy, willing enough to have both her charges under surveillance at once, made no objection. But Delia's presence brought that of Tucker too, who could not keep away from her, and who made all sorts of excuses to slip from his duties to be in the same room with her, though he had no place in her good graces and could scarcely win a glance from her eyes.

He loved her, though he knew it was quite hopeless; and he half hated her sometimes, and was furiously jealous even of Fred, who never looked at her, and of Arthur, who certainly did look at her very often, and who had more than once sent her flowers. Miss Betsy, who could overawe most people, could not frown him out of any room where Delia was.

One wet and stormy afternoon, when custom was very slack, and seemed, indeed, almost to cease, these four people were assembled in the book shop. Mr. Proudie was busy in his own department, and Mrs. Proudie, of whom nobody took very much account, was doubtless slumbering upstairs.

George Tucker had but just joined the four among the books; the little man was in a very evil temper, and took small pains to hide it. Barbara and Fred were busy in different parts of the shop; Miss Betsy sewed with a swift, unremitting energy, but Delia was idle. She sat on a low seat, carried down for her by Fred from the room above; her hands were folded in her lap, her eyes fixed absently on the dull glow of the stove; she had forgotten the storms that beat outside, she saw beautiful visions in the fire's red heart.

She did not look up even at the tap of the cripple's crutch on the floor; it was another region she was in, where rainbow fancy clothes the world and cheats its nakedness into beauty.

"Haven't you a word for a fellow?" cried Tucker, stung into passion by her neglect. "You treat me as if I were a dog!"

"A dog would have better manners," said Delia, coming out of her painted chamber and speaking with dignity.

"Oh, yes, it's our manners you fling up your head at; we ain't good enough for you now—not we, since gentlemen 'ave comed and made their 'omes here. It's mighty condescending of such grand gentlemen to notice us 'umble folks, but if all was known that I know, there's some as wouldn't 'old their 'eads so 'igh——"

Delia got up from her chair; her cheeks were burning, her eyes flashing, her voice vibrated with strong feeling.

"Why are you always so mean and spiteful?" she said. "Do you think I shall care for you more and like you better because you say evil of others? You are wicked, and—and sometimes I think I hate you."

"Don't I know that you hate me," cried the cripple bitterly. "It's the other one you love, the fine gentleman that's playing with you, and laughing at you when he goes away, and sayin' what a precious little fool ——"

But here Miss Betsy, though used to squabbles between the pair, interfered.

"Delia," she commanded, "leave the room, go up to your mother; and you, ye Satan," cried Miss Betsy, driven to the expression, and with whole deeps of righteous wrath in her voice, "maybe now that the lassie's gone ye'll hold your vile tongue."

"I won't hold my tongue," he screamed, scarcely waiting till the door closed behind the trembling Delia. "I ain't done yet. You'll be more tired of me afore I've done, and I won't speak in private, that I won't. *I* ain't got anythink to be ashamed of; let them as has look out for theirselves. You call me names, Miss Betsy, and you treat me as if I was dirt; but there's one here that ain't fit to breathe the same air as George Tucker, that he ain't. I ain't a gaol-bird. I ain't forged nobody's name, and got lagged, and done my five years for it."

His dreadful passion had compelled a hearing; you could have heard a pin drop in the room but for the furious torrent of his words. Fred stood as if he were turned into stone; Barbara was staring with a kind of shrinking horror at the cripple. It was Miss Betsy who spoke.

"If this is true," she began —

Then Fred stepped forward, his face was very white, but he spoke quietly.

"It is quite true," he said, "I am guilty. Years ago I fell, and in a moment of temptation I committed the sin he speaks of, and paid the penalty. Your brother knew." He looked at Miss Betsy. "He took me in the goodness of his heart to give me another chance, but I have suffered a torture under the kindness I have received here, knowing how unworthy I was of it."

"And yet you make up to Barbara!" cried Tucker, his passion not yet all spent, "though you're so 'umble now you're found out, you ain't ashamed to make love."

"No," said Fred, looking at his assailant, "of that I am not guilty. You have a right—the right of every man who has not fallen—to scorn me, but you wrong me there. Love and marriage are not for me. Who would look at me, knowing my story? I will go away from you all now that you know it. I will not pain you or try your kindness by reminding you of all you did for one who—who——"

"Sir," said Miss Betsy with a simple dignity, "your story is safe with Barbara and me, as it was safe with my brother, and you will stay, if you please, till it is his pleasure you should go. We're simple folks, Mr. Eastgate, and it's not for us to judge; if you've repented of your sin as I think you have, it would ill set us to cast it up to you, when there's One above that has promised forgiveness to them that seek it. As for you, ye tale-pyat, that can sneak and listen and find out a man's secrets to expose him, be thankful that your power to work wickedness isn't equal to your will. If ever you open your lips or breathe a word of what you've said this day into mortal ear, its Betsy Proudie ye'll have to reckon with. Out ye go, ye black-hearted scoondrel, an' if I catch as much as a whisper of your ill tongue, it's out ye'll go for guid an' a'." She drove him before her and followed him from the room.

There was a long silence before Fred spoke.

"Miss Barbara," he said, "I tried to tell you—but, you would not listen. And—I was a coward—I was afraid of all I should lose. I have lost it now, and knowing that—will you spare me your scorn?"

"My scorn?"

There was a strange vibrating quiver in her voice; he looked up, his heart beating painfully; there were tears in her eyes.

"You—you forgive me—you do not shudder away from me, now that you know what I have been——"

She held out her hand in answer. Her face was very sad, but it was very tender too.

"Oh," she said, "I have been unkind to you very often, but I did not know. And which of us is good enough to stand aside and say—'I will not forgive my brother who fell by the way, as I too, have fallen, and who has repented and is pardoned, as I, too, hope to be pardened?' Oh, don't think so hardly of me as that!"

"I think—I think you are an angel," said Fred brokenly.

*(To be continued.)*

